# Fluid surface tension evaluation using capillary wave measurement with optical coherence tomography

**DOI:** 10.1063/1.5143935

**Published:** 2020-05-19

**Authors:** Hsiao-Chuan Liu, Piotr Kijanka, Matthew W. Urban

**Affiliations:** 1 Department of Radiology, Mayo Clinic, 200 First St. SW, Rochester, Minnesota 55905, USA; 2 Department of Robotics and Mechatronics, AGH University of Science and Technology, Al. Mickiewicza 30, Krakow 30-059, Poland; 3 Department of Physiology and Biomedical Engineering, Mayo Clinic, 200 First St. SW, Rochester, Minnesota 55905, USA

## Abstract

The surface tension of biological fluids is an important parameter because the mechanical properties of fluids are closely linked with hematological diseases and other pathophysiologies. Capillary waves are associated with fluid mechanical properties. Here, we propose a method that utilizes the acoustic radiation force (ARF) to generate propagating waves and optical coherence tomography (OCT) to measure the wave motion. This ARF-OCT method is capable of evaluating the surface tension of fluids, water and porcine whole blood in this study, based on the dispersion relation of capillary waves. Two-dimensional Fourier transforms were used to decompose frequency components of wave motion images to obtain a *k*-space representation and estimate the wave phase velocity. The phase velocity of capillary waves was obtained from the experimental results and compared to theoretical calculations. The surface tensions of water and porcine whole blood were determined from the experimental results. We first report that capillary waves measured with OCT can be a new promising modality for measuring the surface tension of fluids. The proposed method could be used to differentiate actual pathologic fluids or blood from those taken from healthy subjects and as a biomarker in future biomedical applications.

Surface tension is derived from cohesive forces of molecules pulled equally in every direction by neighboring liquid molecules so that the net force is zero (equilibrium).^1^ Surface tension properties, defined as energy per unit area (dyn/cm) or force per unit length (N/m),^2^ of biological liquids are closely linked with various physiological processes.^3–5^ Pathological processes such as lung carcinogenesis^6^ influence the surface tension of alveolar sacs,^7^ and blood clot formation is associated with the surface tension of blood.^8^ Therefore, the determination of the surface tension of biological fluids could be beneficial for understanding the pathological steps and physiological conditions.^3–5^

The DuNoüy ring method and the Wilhelmy plate method are two classical techniques for measuring the surface tension at a liquid–air interface.^1,2,9^ However, extra correction factors (Harkins and Jordan) and large volumes of studied solutions are limitations of these methods.^2^ The droplet method is also a well-known technique to measure the surface tension, but the limitation to this method is related to the concentration of the surfactants due to the exhibition of relatively weak surface activity.^10^ Kalantarian *et al.* proposed an image analysis method named axisymmetric drop shape analysis (ADSA) to estimate the surface tension.^11^ However, a high image quality is required to be able to define the boundary of drops. Laser-based optical methods such as optical traps^12^ and CO_2_ laser^13^ were proposed to estimate the surface tension of protein condensates in the *μ*N/m scale and soft glass fibers at a high temperature (at least 1000 °C) for industrial applications, respectively. In addition, a He–Ne laser was used to measure the light reflection from the liquid surface to determine the contact angle and surface tension.^14^ For microscopy-based methods, Bertocchi *et al.* determined the surface curvature of drops discriminated by back-reflected light to determine the surface tension.^15^ For both laser-based and microscopy-based methods, a delicate calibration process will always be necessary because the surface curvature is a critical parameter.^15^

Optical coherence tomography (OCT) has been used to evaluate the mechanical properties of solid biological materials due to high resolution, the direct measurement of the wave propagation, and noncontact operation.^16–19^ However, it has not been widely studied to investigate the rheological properties in liquids. Capillary-wave techniques are being developed to obtain more insight into surface properties.^20–23^ Our previous report demonstrates that capillary waves can be generated by an acoustic radiation force (ARF) and monitored by OCT.^24^ In this study, we first report that OCT could be a new modality to evaluate the mechanical property, surface tension, of liquids based on the characterization of the dispersion of capillary waves in frequency dependent rheology. The perturbation of capillary waves can be completely monitored due to a relatively large field-of-view of OCT compared with laser-based methods.^20,23^ In addition, we characterize biological fluid, porcine whole blood, in this paper. Acoustic radiation forces were exerted on the fluid–air interface to generate a capillary wave, which can be imaged similar to watching ripples on a water surface created by dropping a stone in a pond, assuming that the depth of the water is smaller than half of a wavelength.^25^ The theoretical calculations of the surface tension were compared with our experimental results.

In Navier–Stokes theory, the surface tension is associated with the phase velocities of capillary waves *C*_*p*_, fluid depth *d*, and fluid density *ρ*.^4,20,26^ A capillary wave is a surface wave propagating along the interface of fluids between a liquid and air. In this study, we consider a fluid–air free surface with small amplitude waves and a fluid that is assumed to be of uniform depth, incompressible, and irrotational. The wave propagation in the *x*-direction with a phase velocity $C_{p}$ can be described by the following equation:^25^

$$C_{p}=\frac{\omega }{k}=\sqrt{\frac{1-\left(\frac{\rho^{\prime }}{\rho }\right)}{1+\left(\frac{\rho^{\prime }}{\rho }\right)}\left(\frac{g}{k}+\frac{\sigma k}{\Delta \rho }\right)\text{tanh}\left(kd\right)},$$ (1)

where g is the gravity, k is the wavenumber (2π/λ), *d* is the depth of the fluid, ω is the angular frequency, σ is the surface tension, and Δρ is the mass density difference between two media. ρ and $\rho^{\prime }$ are the mass densities of the liquid and air, respectively. The Eötvös (*Eo*) number (or Bond number) is a factor to characterize the regime between the gravity and capillary waves,

$$Eo=\frac{\Delta \rho g}{\sigma k^{2}}=\frac{\Delta \rho g\lambda^{2}}{\sigma {\left(2\pi \right)}^{2}}={\left(\frac{\lambda }{2\pi \lambda_{capillary}}\right)}^{2},$$ (2)

where $\lambda_{capillary}=\sqrt{\sigma /\Delta \rho g}$ is the capillary length and Δρ is the difference in densities between the liquid and air. For a liquid–air interface, if λ is much smaller than $2\pi \lambda_{capillary}$ (Eo ≪ 1), the wave is classified as a capillary wave.

Figure 1 illustrates the theoretical calculation of phase velocities *C*_*p*_ of water and porcine whole blood for capillary waves with Eq. (1). We assume surface tension values of 73 mN/m for water^27^ and 55 mN/m for whole blood.^4^ The mass densities of water and whole blood are 1000 kg/m^3^ and 1060 kg/m^3^, respectively.^28,29^ The mass density of air is 1.2 kg/m^3^, and the gravity is 9.81 m/s^2^. The fluid depth *d* is assumed to be 3 mm. By substituting these parameters into Eq. (1), the theoretical phase velocity of capillary waves in whole blood (red curve) and water (blue curve) can be obtained, as shown in Fig. 1. Therefore, the critical wavelength $\lambda_{c}$ in water and in whole blood was evaluated to be 17.25 mm (blue curve in Fig. 1) and 14.45 mm (red curve in Fig. 1), respectively. The corresponding minimum phase velocity $C_{p\left(min\right)}$ in water and in whole blood was 0.224 m/s and 0.209 m/s, respectively. Once the wavelength is smaller than the critical wavelength, the waves will enter the capillary regime and the gravity is considered to be negligible.^26,30^ The dispersion relation in (1) can be written as

$$\omega^{2}=\frac{1-\left(\frac{\rho^{\prime }}{\rho }\right)}{1+\left(\frac{\rho^{\prime }}{\rho }\right)}\left(gk+\frac{\sigma k^{3}}{\Delta \rho }\right)\text{tanh}\left(kd\right).$$ (3)

Due to the liquid–air interface, the first term on the right side of Eq. (3) is approximately equal to 1 and Δρ is approximately 1000 kg/m^3^. The deep water and shallow water regimes are defined as the regimes where *d* is larger than 0.5λ and smaller than 0.05λ, respectively.^25^ In our case, the depth of fluids was considered to be 3–5 mm and λ was approximately 6 mm (as will be presented later); therefore, the capillary waves are considered to be in the deep water (d>0.5λ) regime in the most cases, and $\text{tanh}\left(kd\right)=\text{tanh}\left(2\pi d/\lambda \right)$ is close to 1. Capillary waves in the shallow water regime are not considered in this study because the depth *d* of the liquid would need to reach to a few hundred micrometers. We rewrite Eq. (3), and the surface tension based on experimental parameters $\sigma_{exp}$ can be evaluated by the following equation:

$$\sigma_{exp}=\frac{\Delta \rho }{k_{exp}^{3}}\left(\omega_{exp}^{2}-gk_{exp}\right),$$ (4)

where

$$\omega_{exp}=\frac{2\pi C_{p\text{\_}exp}}{\lambda_{exp}}.$$ (5)

In practice, $k_{exp}$ can be obtained by the *k*-space calculated by two-dimensional Fourier transform (2D-FT)^31^ of capillary wave motions in the temporal space monitored by OCT. The capillary wave phase velocity $C_{p\text{\_}exp}$ and the surface tension $\sigma_{exp}$ in water and in whole blood can be estimated according to Eqs. (1) and (4), respectively. The details are described in the following paragraphs.

**FIG. 1.**
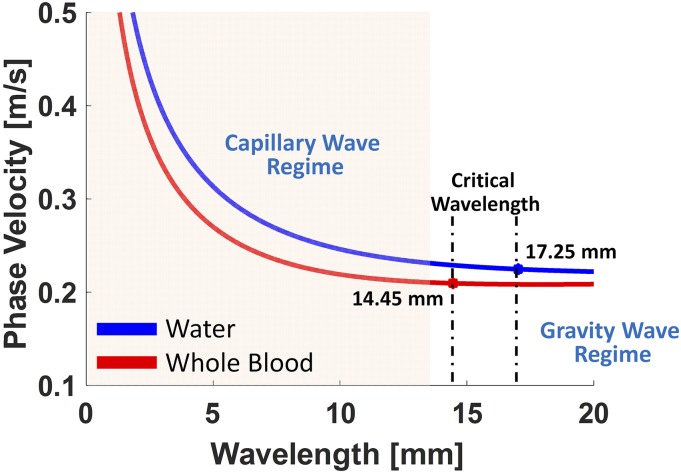
The theoretical calculations of capillary waves on water (blue color) and porcine whole blood (red color) are illustrated. The critical wavelength $\lambda_{c}$ in water was obtained to be 17.25 mm and its minimum phase velocity $C_{p\left(min\right)}$ at the wavelength is approximately 0.224 m/s. The critical wavelength $\lambda_{c}$ in whole blood is 14.45 mm, and the corresponding $C_{p\left(min\right)}$ was calculated to be 0.209 m/s.

The whole blood preparation is followed by a standard protocol.^32^ The porcine whole blood with the K3-EDTA anticoagulant was purchased from LAMPIRE Biological Laboratories, Inc. (Pipersville, PA, USA). The 1 l bottle with porcine whole blood was inverted 5 times gently and then 4 ml of blood was transferred to the Petri dish for the experiment. The bottom of the Petri dish was replaced with a Mylar film. The thickness of the Mylar film is only 100 *μ*m, and the film is acoustically transparent than the plastic of the Petri dish.^33^ Therefore, the acoustic power will be only weakly attenuated. The tap water from our laboratory was used in this study. The depth of fluids was around 3–5 mm. The temperature of water and whole blood was 21 °C (room temperature). The capillary waves on water and porcine whole blood were recorded by using an acoustic radiation force optical coherence tomography (ARF-OCT) system. It was composed of a spectral domain OCT (SD-OCT) scanner with 1300 nm central wavelength (TEL320C1, Thorlabs, Inc., Newton, NJ, USA), a 7.5 MHz focused transducer (ISO703HR, Valpey-Fisher, Hopkinton, MA, USA), three function generators (33250A, Agilent, Santa Clara, CA, USA), and a radiofrequency (RF) amplifier (240L, Electronics and Innovation, Ltd., Rochester, NY, USA), as illustrated in Fig. 2. Three function generators were employed to manage the whole system. Acoustic radiation force was initially generated by function generator 2 and was amplified to 50 dB by a radiofrequency (RF) power amplifier to drive the transducer. Function generator 1 was used to trigger excitation and provide synchronization for the whole system. Function generator 3 was set to control the OCT scan rate at 10 kHz with square pulse trains. More details about the excitation methods can be found in a previous article.^24^

**FIG. 2.**
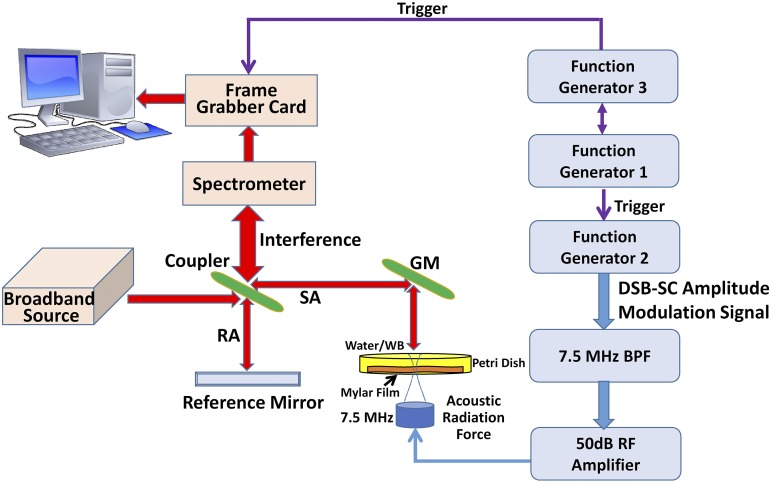
Schematic of the acoustic radiation force optical coherence tomography (ARF-OCT) measurement technique. The three function generators are utilized to provide signals to generate the ARF and synchronize signals with the OCT system. RA: reference arm, SA: sample arm, WB: whole blood, GM: galvanometer mirror, BPF: bandpass filter, and RF: radiofrequency.

The OCT system is capable of producing 13 *µ*m lateral resolution and 3.6 mm of penetration depth within 10 × 10 mm^2^ field of view (FOV) to detect the motion of particles on the surface. The 3.6 mm is provided by Thorlabs, Inc., which is the maximum imaging depth in air that the system displays. The penetration depth is governed by the level of optical scattering and absorption for a given sample. In the whole blood, this depth was on the order of 0.3 mm. To be able to capture the propagation of capillary waves on water and whole blood, the M-B scan mode with 10 kHz scan rate was used to track dynamic processes in the space–time domain. The customized acquisition was set to obtain the data at 100 lateral positions with 0.1 mm interval within a 10 mm FOV and 500 axial scans at 10 kHz scan rate at each position. The acoustic radiation force generated with a 2 ms excitation signal repeated every 50 ms was transmitted to excite the fluids to generate capillary waves. Our previous research reports that the double sideband suppressed carrier amplitude modulation (DSB-SC AM) technique was a useful tool for characterizing the viscoelastic properties of a tissue mimicking a phantom material.^34^ Therefore, it was adopted in the study to create the capillary waves on water and whole blood. In this study, ten cases were collected for water and 20 cases were collected for blood.

The capillary wave motions were determined by the autocorrelation method.^35^ Before autocorrelation, a median filter was employed to remove noise from an original OCT wave motion image. The data in each column of the wave motion image were upsampled by five times by spline interpolation so that the maximum of the motion signal and the time of that peak can be found at each spatial location. After the autocorrelation calculation, spatiotemporal wave motion images were reconstructed, as shown in Fig. 3(a) for water and Fig. 3(b) for the porcine whole blood, with white dashed pentagons. A 2D-FT was used to decompose multiple frequencies from wave motion images of water and whole blood to obtain a *k*-space illustrated in Figs. 3(c) and 3(d), respectively.^31^

**FIG. 3.**
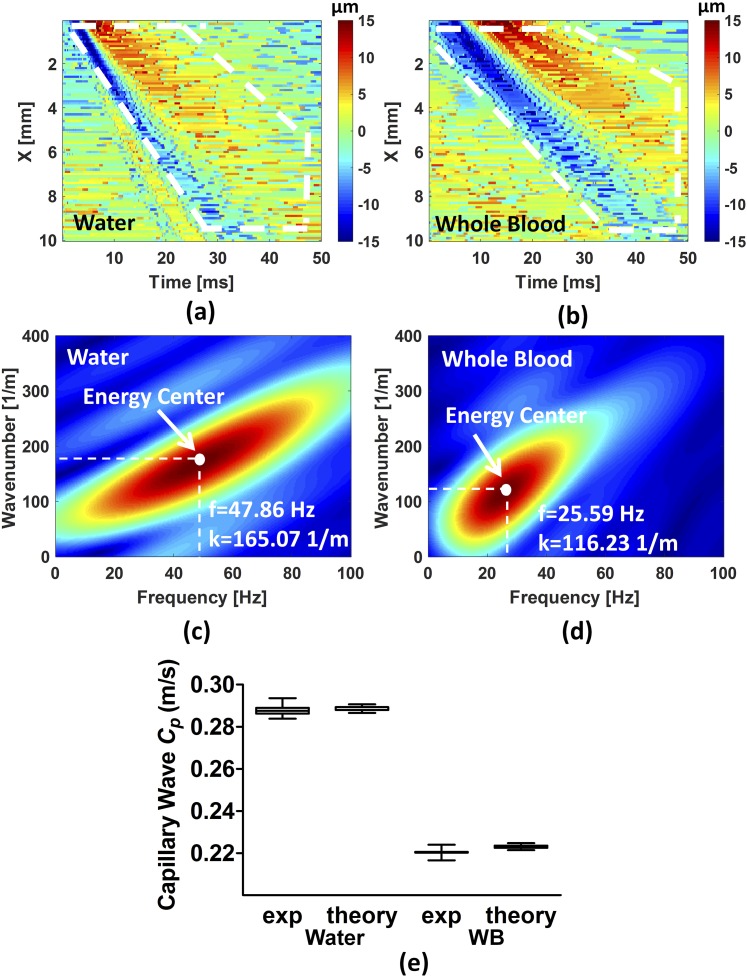
A 2D-OCT wave displacement images on water (a) and porcine whole blood (b) are illustrated. The capillary waves (white dashed pentagon) were clearly observed. The *k*-space of the capillary wave motion in water (c) and in porcine whole blood (d) is displayed as an example. (e) Boxplots of the phase velocities of capillary waves in water and whole blood were theoretically and experimentally determined. The letters “*exp*” on the horizontal axis represents the results from experiments.

To determine the surface tension of water and whole blood, wavenumbers $k_{exp}$ and capillary wave phase velocities $C_{p\text{\_}exp}$ need to be determined. The maximum energy peak in the *k*-space, as shown in Fig. 3(c) for water and Fig. 3(d) for whole blood, was selected to determine the corresponding $k_{exp}$ and frequency coordinates to determine $C_{p\text{\_}exp}$. Figures 3(c) and 3(d) illustrate an example of a wavenumber and its corresponding dispersion frequency in capillary wave on water and whole blood. The capillary wave phase velocities can be determined by a single dispersion frequency divided by its associated wavenumber. Figure 3(e) demonstrates the experimental and theoretical results of the phase velocity in the capillary wave on water and whole blood.

All the parameters that were assumed to construct the curves in Fig. 1 are assumed here. For the theoretical calculation, we assumed the values of surface tension of 73 mN/m for water^27^ and 55 mN/m for whole blood.^4^ The mass densities of water and whole blood are 1000 kg/m^3^ and 1060 kg/m^3^, respectively.^28,29^ The mass density of air is 1.2 kg/m^3^ and gravity is 9.81 m/s^2^. The fluid depth *d* is assumed to be 3 mm. According to Eq. (1), the theoretical phase velocities with various wavelengths from 0 mm to 20 mm were calculated and illustrated in Fig. 1. Based on Fig. 1, the theoretical capillary phase velocity is 0.2891 m/s for water at the measured wavelength of 6.1 mm (or wavenumber of 165.07 m^−1^) and 0.2262 m/s for whole blood at the measured wavelength of 8.6 mm (or wavenumber of 116.23 m^−1^). These wavelengths were identified from the energy center of the *k*-space, as they represent the primary wave propagating on the surface of the fluid.

For the experimental calculation, the experimental phase velocity is calculated by the frequency divided by the wavenumber at the energy center of a *k*-space. According to Fig. 3(c), the experimental phase velocity is 0.2899 m/s for water and 0.2201 m/s for whole blood according to Fig. 3(d). The accuracy of the phase velocity is 99.72% for water and 97.3% for whole blood. This accuracy evaluation is valid with respect to the reported literature values for the surface tension and density and for the measured wavelengths.

We performed ten replicate measurements in water and 20 replicate measurements in whole blood. The reason that more measurements were made in the blood is possible inhomogeneity in the blood compared to the water (for testing the system) and to produce more reliable statistical results for the biological case. The phase velocities in experimental and theoretical calculations were 0.28 ± 0.0026 m/s and 0.28 ± 0.0011 m/s for water, respectively, and 0.22 ± 0.002 m/s and 0.22 ± 0.0008 m/s for porcine whole blood, respectively. The standard deviations in the theoretical results in Fig. 3(e) are due to slight shifting of energy center in each measured *k*-space realization. In this study, we observed very close agreement and high accuracy of phase velocities between experimental and theoretical results in both water and whole blood.

According to Eq. (4), the surface tension is directly proportional to $C_{p\text{\_}exp}^{2}$ and inversely proportional to $k_{exp}^{3}$. Figure 4 clearly exhibits that the surface tension in water is larger than that in whole blood, which matches our results that the phase velocities in water are more than those in whole blood, as shown in Fig. 3(d). Figure 4 shows that the mean values of surface tension are 71.26 ± 1.22 mN/m for water and 51.14 ± 1.34 mN/m for whole blood. A well-known work published by Vargaftik *et al.* reported that the surface tension of water at 20 °C is 72.75 mN/m.^36^ The surface tension of distilled water at 22 °C is 72.45 mN/m.^4^ Takamura *et al.* described that the salinity at 20 °C is approximately 73 dyn/cm.^27^ On the other hand, the surface tension of human whole blood at 22 °C was reported to be approximately 55 mN/m.^4^ Our results have very good agreement with previous studies.

**FIG. 4.**
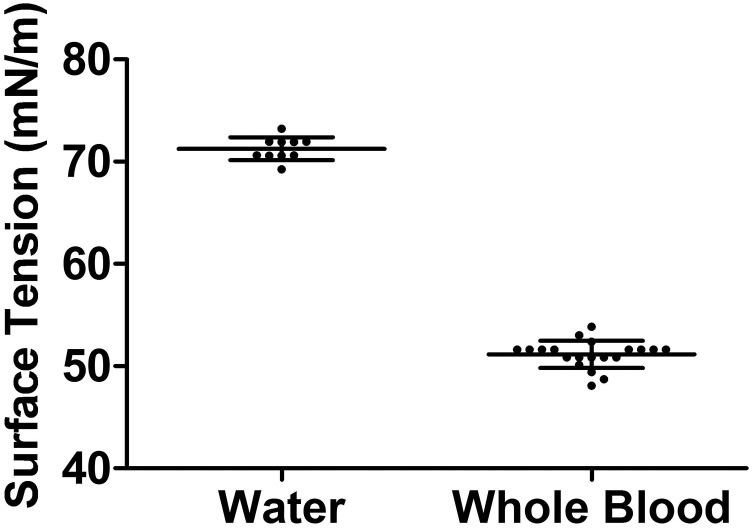
Boxplots of the surface tension was displayed based on the maximum energy peak of capillary waves in the *k*-space.

In summary, we demonstrate that the capillary waves-based method ARF-OCT might be a promising tool to evaluate the surface tension of biological fluids. The acoustic radiation force is a useful noncontact method to generate capillary waves on the surface of fluids, which can be recorded by OCT. The capillary wave phase velocities from the theory and experiments revealed significant agreement. Human biological fluids such as mucus, cerebrospinal fluids, endocrine glands, and alveolar lining fluids contain considerable surfactants and proteins.^37^ Various molecular weight surfactants control the surface tension in tissues of the human body. The surface tension behavior is an important physicochemical feature affected by various disorders.^37,38^ Changes in the surface tension behavior of biological fluids are associated with certain disease and its severity, such as respiratory distress syndrome, glomerulonephritis, chronic bronchitis, and neurosyphilis.^37^ The ARF-OCT would be a promising tool to evaluate the surface tension in various biological fluids and hematological diseases and a useful biomarker in the future biological applications.

## References

[1] A. Ferguson, J. Sci. Instrum. 10(2), 34–37 (1933). 10.1088/0950-7671/10/2/302

[2] Handbook of Adhesives and Surface Preparation, edited by Ebnesajjad S. (William Andrew Publishing, Oxford, 2011), pp. 21–30.

[3] D. V. Trukhin, O. V. Sinyachenko, V. N. Kazakov, S. V. Lylyk, A. M. Belokon, and U. Pison, Colloids Surf., B 21(1-3), 231–238 (2001). 10.1016/s0927-7765(01)00175-8 11377951

[4] E. Hrncir and J. Rosina, Physiol. Res. 46(4), 319–321 (1997). 9728499

[5] H. N. Harkins and W. D. Harkins, J. Clin. Invest. 7(2), 263–281 (1929). 10.1172/jci100228 16693861 PMC434787

[6] X. Wang, L. Wang, H. Zhang, K. Li, and J. You, Oncol. Lett. 12(6), 4357–4360 (2016). 10.3892/ol.2016.5259 28101199 PMC5228324

[7] S. Subramaniam, P. Bummer, and C. G. Gairola, Fundam. Appl. Toxicol. 27(1), 63–69 (1995). 10.1093/toxsci/27.1.63 7589929

[8] K. Mccuaig, C. W. Lloyd, J. Gosbee, and W. W. Snyder, Am. J. Surg. 164(2), 119–123 (1992). 10.1016/s0002-9610(05)80368-x 1636890

[9] P. L. du Noüy, J. Gen. Physiol. 7(5), 625–631 (1925). 10.1085/jgp.7.5.625 19872165 PMC2140742

[10] T. Kairaliyeva, E. V. Aksenenko, N. Mucic, A. V. Makievski, V. B. Fainerman, and R. Miller, J. Surfactants Deterg. 20(6), 1225–1241 (2017). 10.1007/s11743-017-2016-y 29200810 PMC5686271

[11] A. Kalantarian, S. M. I. Saad, and A. W. Neumann, Adv. Colloid Interface Sci. 199-200, 15–22 (2013). 10.1016/j.cis.2013.07.004 24018120

[12] L. M. Jawerth, M. Ijavi, M. Ruer, S. Saha, M. Jahnel, A. A. Hyman, F. Julicher, and E. Fischer-Friedrich, Phys. Rev. Lett. 121(25), 258101 (2018). 10.1103/physrevlett.121.258101 30608810

[13] K. Boyd, H. Ebendorff-Heidepriem, T. M. Monro, and J. Munch, Opt. Mater. Express 2(8), 1101–1110 (2012). 10.1364/ome.2.001101

[14] D. Luo, L. Qian, L. Dong, P. Shao, Z. Yue, J. Wang, B. Shi, S. Wu, and Y. Qin, Opt. Express 27(12), 16703–16712 (2019). 10.1364/oe.27.016703 31252892

[15] C. Bertocchi, A. Ravasio, S. Bernet, G. Putz, P. Dietl, and T. Haller, Biophys. J. 89(2), 1353–1361 (2005). 10.1529/biophysj.104.053132 15951375 PMC1366620

[16] K. S. Yemul, A. M. Zysk, A. L. Richardson, K. V. Tangella, and L. K. Jacobs, Surg. Innov. 26(1), 50–56 (2019). 10.1177/1553350618803245 30295149

[17] K. V. Larin and D. D. Sampson, Biomed. Opt. Express 8(2), 1172–1202 (2017). 10.1364/boe.8.001172 28271011 PMC5330567

[18] Y. Qiu, Y. Wang, Y. Xu, N. Chandra, J. Haorah, B. Hubbi, B. J. Pfister, and X. Liu, Biomed. Opt. Express 7(2), 688–700 (2016). 10.1364/boe.7.000688 26977372 PMC4771481

[19] S. Wang and K. V. Larin, J. Biophotonics 8(4), 279–302 (2015). 10.1002/jbio.201400108 25412100 PMC4410708

[20] C. Cinbis and B. T. Khuri-Yakub, Rev. Sci. Instrum. 63(3), 2048–2050 (1992). 10.1063/1.1143164

[21] A. Shmyrov, A. Mizev, A. Shmyrova, and I. Mizeva, Phys. Fluids 31(1), 012101 (2019). 10.1063/1.5060666

[22] D. Langevin, Colloids Surf. 43(2-4), 121–131 (1990). 10.1016/0166-6622(90)80284-b

[23] F. Zhu, R. Miao, C. Xu, and Z. Cao, Am. J. Phys. 75(10), 896–898 (2007). 10.1119/1.2750379

[24] H.-C. Liu, P. Kijanka, and M. W. Urban, Biomed. Opt. Express 11(2), 1092–1106 (2020). 10.1364/boe.382819 32206401 PMC7041467

[25] H. Lamb, Hydrodynamics (Cambridge University Press, 1932).

[26] F. Behroozi, J. Smith, and W. Even, Am. J. Phys. 78(11), 1165–1169 (2010). 10.1119/1.3467887

[27] K. Takamura, H. Fischer, and N. R. Morrow, J. Pet. Sci. Eng. 98-99, 50–60 (2012). 10.1016/j.petrol.2012.09.003

[28] R. J. Trudnowski and R. C. Rico, Clin. Chem. 20(5), 615–616 (1974). 10.1093/clinchem/20.5.615 4826961

[29] D. J. Vitello, R. M. Ripper, M. R. Fettiplace, G. L. Weinberg, and J. M. Vitello, J. Vet. Med. 2015, 4 10.1155/2015/152730 PMC4590883 26464949

[30] Y. V. Sanochkin, Fluid Dyn. 35(4), 599–604 (2000). 10.1007/bf02698130

[31] M. Bernal, I. Nenadic, M. W. Urban, and J. F. Greenleaf, J. Acoust. Soc. Am. 129(3), 1344–1354 (2011). 10.1121/1.3533735 21428498 PMC3078026

[32] O. Linderkamp, H. T. Versmold, K. P. Riegel, and K. Betke, Pediatrics 74(1), 45–51 (1984). 6204271

[33] J. Lee, S.-Y. Teh, A. Lee, H. H. Kim, C. Lee, and K. K. Shung, Ultrasound Med. Biol. 36(2), 350–355 (2010). 10.1016/j.ultrasmedbio.2009.10.005 20045590 PMC2815109

[34] M. W. Urban, M. Fatemi, and J. F. Greenleaf, J. Acoust. Soc. Am. 127(3), 1228–1238 (2010). 10.1121/1.3294487 20329821 PMC2856505

[35] M. L. Palmeri, M. H. Wang, J. J. Dahl, K. D. Frinkley, and K. R. Nightingale, Ultrasound Med. Biol. 34(4), 546–558 (2008). 10.1016/j.ultrasmedbio.2007.10.009 18222031 PMC2362504

[36] N. B. Vargaftik, B. N. Volkov, and L. D. Voljak, J. Phys. Chem. Ref. Data 12(3), 817–820 (1983). 10.1063/1.555688

[37] A. Fathi-Azarbayjani and A. Jouyban, Bioimpacts 5(1), 29–44 (2015). 10.15171/bi.2015.31 25901295 PMC4401165

[38] G. P. Topulos, R. E. Brown, and J. P. Butler, J. Appl. Physiol. 93(3), 1023–1029 (2002). 10.1152/japplphysiol.00779.2001 12183499

